# Supplementary material for: Identification of photoperiod-induced specific miRNAs in the adrenal glands of Sunite sheep (Ovis aries)
Source: Front Vet Sci. 2022 Jul 22;9:888207. doi: 10.3389/fvets.2022.888207 (PMC9354845; doi:10.3389/fvets.2022.888207)
Supplement: Supplementary file 1 [file Data_Sheet_1.ZIP › Supplementary file/miRDeep2 file.zip!/miRDeep2 file/Novel_Structure/LP42A1/Novel_416.pdf]

Provisional ID	:	NC_019458.2_257
Score total	:	2.2
Score for star read(s)	:	-1.3
Score for read counts	:	0
Score for mfe	:	1.9
Score for randfold	:	1.6
Score for cons. seed	:	
Total read count	:	35
Mature read count	:	35
Loop read count	:	0
Star read count	:	0

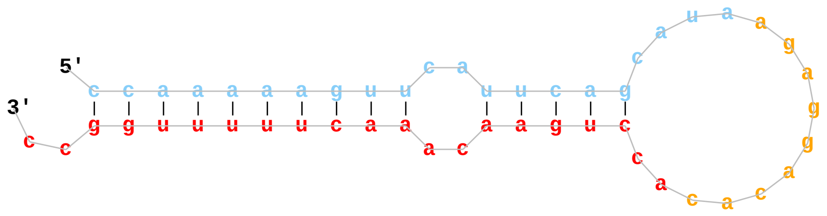

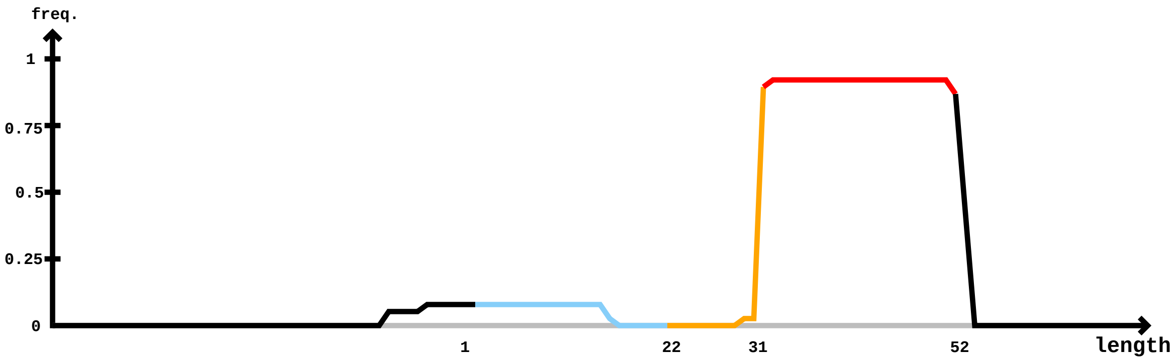


```
                                                Star                            Mature
5'- aucacauuagugaagugcuaucccauagaaauauuggguuggccaaaaaguucauucagcauaagaggacacaccugaacaaacuuuuuggccaacccaguauaaagcaagc   -3'    exp
    .((((....))))..((((...........((((((((((((((((((((((..(((((...............)))))..))))))))))))))))))))))..))))...    reads      mm         sample
    .................................uuAgguuggccaaaaaguucauu........................................................    1          1          seq
    .................................uuggguuggccaaaaGguucauu........................................................    1          1          seq
    .....................................Uuuggccaaaaaguucauuc.......................................................    1          1          seq
    ......................................................................acaccugaacaaacuuuuuggc....................    1          0          seq
    ........................................................................aUcugaacaaacuuuuuggcc...................    25         1          seq
    ........................................................................aUcugaacaaacuuuuuggcca..................    8          1          seq
    .........................................................................Ucugaacaaacuuuuuggc....................    1          1          seq
```
